# Copper Dyshomeostasis in Neurodegenerative Diseases—Therapeutic Implications

**DOI:** 10.3390/ijms21239259

**Published:** 2020-12-04

**Authors:** Grażyna Gromadzka, Beata Tarnacka, Anna Flaga, Agata Adamczyk

**Affiliations:** 1 Collegium Medicum, Faculty of Medicine, Cardinal Stefan Wyszynski University, Wóycickiego 1/3 Street, 01-938 Warsaw, Poland; a.flaga@uksw.edu.pl; 2 Department of Rehabilitation, Eleonora Reicher National Institute of Geriatrics, Rheumatology and Rehabilitation, Rehabilitation Clinic, Medical University of Warsaw, Spartańska 1 Street, 02-637 Warsaw, Poland; beatek_ta@poczta.onet.pl; 3 Department of Cellular Signalling, Mossakowski Medical Research Centre, Polish Academy of Sciences, 5 Pawińskiego Street, 02-106 Warsaw, Poland; agataadamczyk72@gmail.com

**Keywords:** Alzheimer’s disease, astrocytes, copper, neurodegeneration, neurons, Parkinson’s disease, treatment, Wilson’s disease

## Abstract

Copper is one of the most abundant basic transition metals in the human body. It takes part in oxygen metabolism, collagen synthesis, and skin pigmentation, maintaining the integrity of blood vessels, as well as in iron homeostasis, antioxidant defense, and neurotransmitter synthesis. It may also be involved in cell signaling and may participate in modulation of membrane receptor-ligand interactions, control of kinase and related phosphatase functions, as well as many cellular pathways. Its role is also important in controlling gene expression in the nucleus. In the nervous system in particular, copper is involved in myelination, and by modulating synaptic activity as well as excitotoxic cell death and signaling cascades induced by neurotrophic factors, copper is important for various neuronal functions. Current data suggest that both excess copper levels and copper deficiency can be harmful, and careful homeostatic control is important. This knowledge opens up an important new area for potential therapeutic interventions based on copper supplementation or removal in neurodegenerative diseases including Wilson’s disease (WD), Menkes disease (MD), Alzheimer’s disease (AD), Parkinson’s disease (PD), and others. However, much remains to be discovered, in particular, how to regulate copper homeostasis to prevent neurodegeneration, when to chelate copper, and when to supplement it.

## 1. Introduction

Copper (Cu) is a generally used heavy metal [1] and is one of the most abundant transition metals essential for survival in the human body [2]. It takes part in several physiological processes, including oxygen metabolism, skin pigmentation, and collagen synthesis, maintaining the integrity of blood vessels, as well as in iron homeostasis, antioxidant defense, and the synthesis of neurotransmitters [3,4,5].

Copper’s role in various physiological processes is due in part to its activity as a cofactor or structural component in various copper proteins. Its oxidation state can change from Cu^+^ to Cu^2+^ [3]; due to this feature, copper acts as a cofactor of enzymes with oxidoreductase activity. Copper-containing amine oxidases catalyze the oxidative deamination of primary amines; it oxidizes tyrosine and dopamine to aldehydes and tyrosinase, participating in the formation of melanin [5,6,7,8]. Mitochondrial cytochrome c oxidase is a copper-containing enzyme complex that receives electrons from cytochrome c and uses them to convert molecular oxygen into water [5,8]. Ceruloplasmin (Cp), which exhibits copper-dependent oxidase activity, converts Fe^2+^ divalent iron in Fe^3+^, and this process is important for transferrin-mediated transport of iron in the plasma [9,10]; in that manner, copper takes part in iron homeostasis. Due to its potent redox effects, copper can also participate in cell signaling and in the modulation of membrane receptor–ligand interactions, can control kinase and related phosphatase functions, as well as participates in many cellular pathways; its role is also important for the control of gene expression in the nucleus [10]. Particularly within the nervous system, copper is involved in myelination and has been shown to play a key role in synapses by interacting with synaptic proteins and neurotransmitter receptors [11,12]. By modulating synaptic activity as well as excitotoxic cell death and neurotrophic-induced signaling cascades, copper is important for a variety of neuronal functions.

Current data suggest that both excessive copper levels and its deficiency may be harmful, and precise homeostatic control is important [3,13,14]. Abnormal copper homeostasis may be the result of genetic mutation, aging, or environmental influences and leads to a variety of pathological sequelae including cancer, inflammation, and neurodegeneration [15,16,17].

This knowledge opens up an important new area for potential therapeutic interventions based on copper supplementation or removal in neurodegenerative diseases. However, much remains to be discovered, in particular, how to regulate copper homeostasis to prevent neurodegeneration, when should copper be chelated, and when it should be supplemented. This question can be answered by looking at copper-dependent neurotoxicity mechanisms and factors that can modulate copper neurotoxicity.

Our review will focus on the role of copper dyshomeostasis in the pathogenesis of neurodegeneration. The usefulness of therapeutic agents that reduce copper-related neurotoxicity in the treatment of neurodegenerative diseases including Wilson’s disease (WD), Menkes disease (MD), Alzheimer’s disease (AD), Parkinson’s disease (PD), prion disease, and others will also be discussed.

## 2. Copper Metabolism in the Human Body

The source of copper for the human body is diet. The average recommended daily intake for Cu is 1 mg. A lot of copper is found in seafood, chocolate, nuts, mushrooms, red wine, leafy greens, legumes, and whole grain cereal products. It is absorbed in the proximal part of the small intestine. Copper absorption facilitates its binding to amino acids (Cys, His, Asp, Met, Tyr, Gly, and Thr), as well as its presence in the form of ligands with organic acids (gluconic, lactic, citric, and vinegar) [18]. Some prebiotics such as pectin, inulin, and fructro-oligosaccharides have been documented to positively affect copper absorption. Copper absorption is reduced by iron, zinc, calcium, molybdenum, phosphorus, and vitamin C [19]. Most of the copper in food is in the oxidized form of Cu^2+^ and must be reduced to Cu ^+^ to be effectively absorbed by the digestive system. Proteins involved in this process are cytochrome b (558) reductase (Dctyb) Fe^3+^/Cu^2+^, Steap 2 (transmembrane epithelial prostate 6), and Cybrd1 (cytochrome b reductase 1), which can reduce copper ions [20]. Most probably, the Cu^+^ ion is transported to the enterocytes by copper transporter 1 (CTR1) that was proposed to serve as an apical copper transporter responsible for copper absorption from the lumen of the gastrointestinal tract [21], as a membrane transporter carrying copper from the bloodstream into the enterocyte, and as an intracellular copper transporter [22,23,24]. Since CTR1 is not able to transport bivalent copper, some of the consumed Cu (II) is probably absorbed by the cells through divalent metal transporter 1 (DMT1) [19,20]. Alternative copper absorption pathways have also been proposed, including endocytosis, anion exchange, and sodium-dependent amino acid transport [25,26].

In duodenal specimens taken from patients with an inherited defect of P-type adenosine triphosphatase (ATPase) 7B (ATP7B) manifested by the phenotype of Wilson’s disease (WND) (OMIM #277900) resulting from body copper overload, CTR1 expression occurred mainly near the apical membrane of the enterocytes. In this study, decreased transcription activity of CTR1 and low CTR1 protein production in WND patients were documented and may be a plausible adaptative mechanism aimed to reduce copper influx into enterocytes and to prevent its overload resulting from disturbed mechanisms of copper biliary excretion [22,24,25]. Therefore, changes in CTR expression in enterocytes may be part of the mechanism regulating the process of copper absorption in the small intestine and preventing copper overload.

Another mechanism may be based on the activity of ATP7B that, as reported by Pierson et al., may modify the intestinal copper absorption mainly by vesicular sequestration of copper within the cell [27]. It has been suggested that, under conditions of excess copper, this metal can be sequestered by ATP7B and safely stored in enterocytes to be available when cytosolic levels decline due to insufficient dietary content.

A protein sharing structural and functional similarities with ATP7B is ATP7A. Most probably, this protein plays a role in copper efflux from enterocytes to the basolateral space rather than in the sequestration of copper into vesicles as it shows highly polarized basolateral localization, which reflects its role in copper efflux to circulation [27,28,29,30]. An important role of ATP7A is well illustrated by the phenotype of Menkes disease (OMIM #30940) [31]. This inherited defect of ATP7A leads to impaired copper transport in the intestinal epithelium resulting in overall copper deficiency [32]. The intestinal expression of ATP7A proteins increases in response to copper overload [27]. In the aforementioned study of WND patients, diminished duodenal expression of CTR1 was accompanied by the increased expression of ATP7A [33]. In the intestines of suckling rat pups fed with copper-rich milk, ATP7A mRNA and protein levels were significantly higher in comparison with animals on a standard diet [34]. Increased ATP7A expression was accompanied by increased copper retention in the intestine of the young rats. Thus, it seems likely that the increased production of ATP7A in the intestine of WND patients serves to retain copper in enterocytes and/or to stimulate its outflow from cells. These observations indicate that, when there is sufficient or excess copper in the body, various copper-sensitive mechanisms are triggered to prevent the body from becoming overloaded with copper. These mechanisms aim for regulation of the expression of copper transporters CTR1, ATP7A, and ATP7B in enterocytes. One of such mechanisms may be related to the activity of a transcription factor Sp1 that is able to bind to the GC boxes located near the CTR1 promoter [35,36]. Sp1 is regulated by copper concentration: in copper sufficiency conditions, copper is bound to Sp1, which prevents its binding to the CTR1 and Sp1 promoters, and oppositely, low copper levels upregulate Sp1 and CTR1 expression. Several Sp-like binding sites were also detected in the promoter of a murine ATP7A orthologue [37]. These data suggest that Sp1 is a candidate regulator of copper homeostasis in humans.

**Figure 1:**
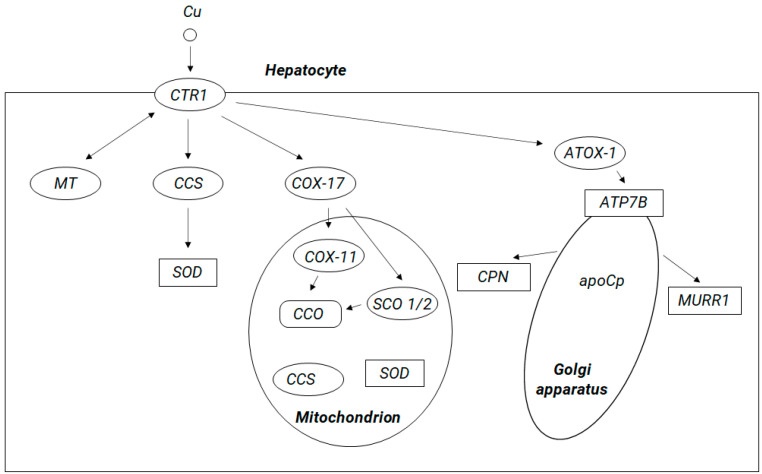
Copper transport paths ([39] with modifications).

As it was mentioned above, from the enterocytes, copper enters the bloodstream via the protein adenosine triphosphatase 7A (ATP7A). Copper enters to the cells via the CTR1 membrane protein. Within human cells Cu^+^ is sequestered by the a ubiquitous cysteine-containing tripeptide glutathione (GSH) and stored in metallothioneins (MTs), both of which are rich in thiol groups, showing high affinity for copper; alternatively, it may be shuttled by copper chaperones to its specific targets. Copper transporting metallochaperones can escort Cu^+^ from CTR1 in the cytosolic pool to facilitate its supply to the specific target compartments. These include cytochrome C oxidase copper chaperone COX17, which delivers copper to mitochondria, copper chaperone for copper, zinc superoxide dismutase (CuZn-SOD, SOD1) (CCS), and antioxidant protein 1 (Atox1), which delivers copper to specific enzymes, molecules, the nucleus, and endoplasmic reticulum (ER) and to ATP7A and ATP7B (see Figure 1). It has been suggested that copper may be directly transferred from CTR1 to chaperones and then to SOD1 via formation of a CTR1-CCS-SOD1 complex [38]. Possibly, this mechanism may exist for other copper metallochaperones. Within cells, ATP7A and ATP7B are responsible for regulating the concentration of copper ions and are involved in the copper transport between intracellular compartments across cellular membranes in an ATP-dependent manner, and thus, they supply copper for incorporation into copper-dependent enzymes such as tyrosinase, peptidylglycine amidating monooxygenase, dopamine monooxygenase, lysyl oxidase, and ceruloplasmin [23]. At high intracellular copper concentration, ATP7A and ATP7B are translocated reversibly to the plasma membrane (ATP7A typically to the basolateral and ATP7B to the apical surface) to facilitate cellular copper export [16]. 

**Figure 2:**
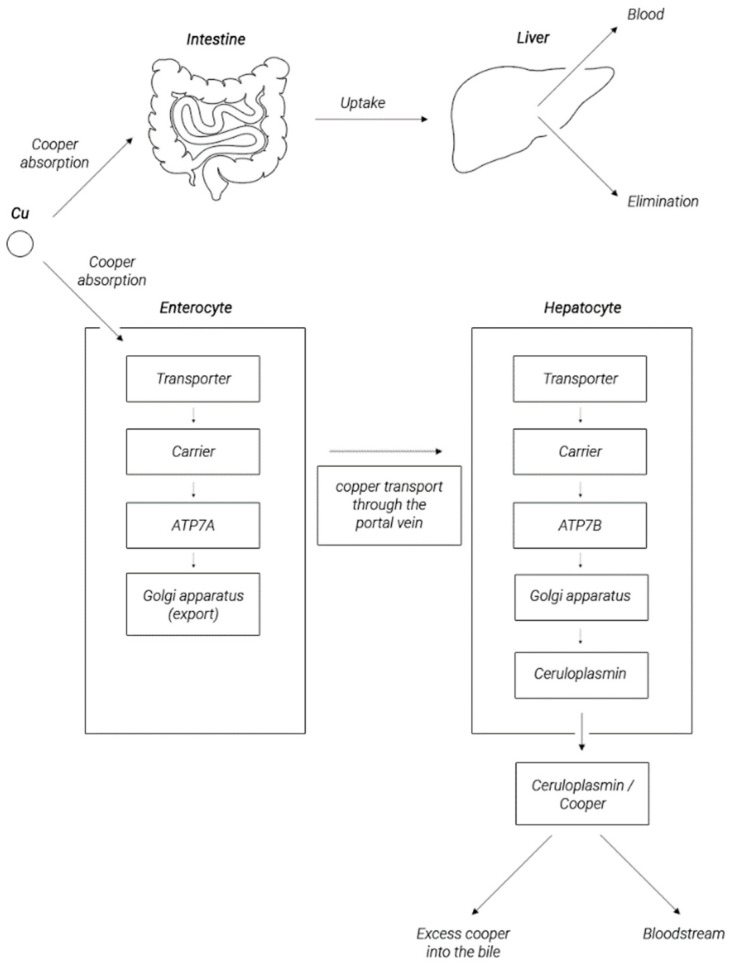
Copper metabolism in the human body (according to [51], modified).

The main organ responsible for metabolism of copper is the liver, which stores most of this element. In response to the elevated copper levels, ATP7B in hepatocytes travels into the vesicles near the apical membrane to transfer excess copper to the bile [23,27]. A significant part of copper is incorporated into the Cp molecule (through a process dependent on ATP7B) and then released into circulation in this form, as shown in Figure 2 [40,41,42,43]. In circulation, copper is not present in the free form, but if not bound to ceruloplasmin, it is associated with proteins, peptides, or amino acids. In this form, this element is distributed with blood throughout the body [22,44]. The normal adult concentration range for copper in sera is between 5 and 25 μM. Copper, complexed with Cp, constitutes 85–95% of the plasma copper pool [45]. The remaining approx. 0.2–1.6 μM (the so-called non-ceruloplasmin copper) is a pool of bioavailable copper and is exchangeable between albumin, α2-macroglobulin, peptides, amino acids, reaching organs, and tissues, including the heart, kidneys, and some others organs presented in Table 1 and the brain. Excess copper is removed with bile. In this way, 98% of the copper is removed from the body, and another 2% is removed in urine [46,47,48,49,50].

## 3. Copper Content in the Brain

The brain is the second organ, after the liver, which accumulates the largest amount of copper [52]. Copper is available to brain cells from blood or cerebrospinal fluid (CSF). The normal range of serum copper concentrations in adults is 5–25 µM, of which approximately 95% is bound to Cp and other major copper binding proteins [45,53]. The concentration of copper in the cerebrospinal fluid (approximately 70–80 µM) is quite high compared to the serum (12–24 µM), which increases the possibility of specific copper signaling in the brain [13,54]. The human brain needs copper for metabolic purposes [29]. The copper content in the brain ranges from 3.1 to 5.1 g/g wet weight [55,56]. Within the brain, the highest concentrations of Cu were measured in the substantia nigra. High levels of copper have also been detected in the cerebellum, hippocampus, hypothalamus, olfactory bulb, and cortex [13,53]. Structures with the highest average concentration of copper in different brain areas, as studied by Borilla [13], are shown in Table 2 (copper concentrations in the hippocampus, cerebellum, frontal cortex, superior temporal gyrus, middle temporal gyrus, midbrain, and pons were not measured in this study).

## 4. Copper Role in the Brain

Over the last 30 years, there has been an increasing interest in the participation of copper in brain function and pathophysiology [13,57]. While the role that copper plays in the brain has not yet been fully elucidated, some progress has been made on this topic. Copper has been documented to be important in many physiological processes in the brain. These processes include the following:

(1) Development of the central nervous system (CNS)—trace amounts of copper are necessary for the performance of functions necessary the proper development of the brain [58];

(2) Functioning of the active site of many enzymes, including dopamine β-hydroxylase-like monooxygenase, involved in the production of norepinephrine [41,59,60];

(3) Mitochondrial activity—cytochrome c mitochondrial oxidase is a copper-containing enzyme complex [5,8];

(4) Biosynthesis of neurotransmitters—copper-containing amine oxidases catalyze the oxidative deamination of primary amines to aldehydes, and tyrosinase oxidizes tyrosine and dopamine, participating in the formation of melanin [5,8];

(5) Defense against oxidative stress—copper acts as a cofactor of enzymes with oxidoreductase activity, including Cu/Zn superoxide dismutase (Cu-Zn SOD), which catalyzes the process of dismutation of superoxide radicals to oxygen and hydrogen peroxide;

(6) Modulation of amino acid receptors and purine receptors [61];

(7) Synaptic transmission—copper is located at the synaptic terminals and can be released by depolarization. It has been documented that the control of copper release into the synapse is critical to modulate synaptic transmission and is controlled by ATP7A at the synapse [62,63,64]. Cu may play a protective role against N-methyl-D-aspartate (NMDA)-mediated excitotoxicity as it has been shown to exert an inhibitory effect on the α-amino-3-hydroxy-5-methyl-4-isoxazolpropionic acid receptor (AMPAR) in addition to a blocking effect on NMDAR. Copper can also control synaptic transmission and related signaling by modulating Ca^2+^ or zinc binding and by regulating metallothionein (MT) expression [65];

(8) Redox signaling—the labile copper pool is believed to be associated with redox signaling. Labile copper was found in the soma of the cerebellar granules and the pyramidal neurons of the cerebral cortex in addition to neuropil in the cerebellum and cortex, hippocampus, and spinal cord [13,66];

(9) Processing of information in the brain—Chang suggested a diagram that includes labile, neuronal copper pools in the Golgi compartment as the source, signal propagation through the postsynaptic membrane receptor/ion channel target (Cu (I) Ctr1 transporter), and copper-dependent spontaneous activity neural network [13,67]. Vesicular storage of neurotransmitters with copper has also been described, suggesting their simultaneous release. Copper cell signaling between neurons and astrocytes has been documented to exist and may play an important role in information processing in the brain [13,68,69];

(10) Modulation of the action of brain-derived neurotrophic factor (BDNF) and nerve growth factor (NGF)—it has been observed that copper has a contrasting, inhibitory, or stimulating effect on the process of neuronal proliferation induced by BDNF or NGF [48,49,50,51]. The molecular basis of these effects is not yet fully understood. It has been documented that copper can interact with the N-terminal domain of BDNF and thus affect its conformation, as well as the binding of BDNF to the receptor on neurons: tropomyosin B receptor kinase [70,71,72]. Copper–NGF interrelation seems complicated. It has been documented that NGF can stimulate copper accumulation in cells and induce copper-dependent methylation of proteins or neurite outgrowth; on the other hand, it has been established that copper can prevent NGF induction of neuroprotection from oxidative damage. These effects may possibly depend on cell type and copper concentration as well as on the influence of copper on various processes, including oxidative stress, protein methylation, or neurite hyperplasia [73,74,75].

Circulating copper enters cells through the membrane protein called copper transporter 1 (CTR1). Inside the cell, copper can (1) remain in the cytosol in a form bound to metallothioneins (MT) or glutathione, (2) be incorporated via the copper chaperone for copper, zinc superoxide dismutase (Cu,Zn-SOD, SOD1 (CCS)) into the cytoplasmic enzyme SOD1, which protects cells from free radical damage, (3) be transferred to the mitochondria (with participation of the chaperone COX17 (chaperone for cytochrome c oxidase)) and be incorporated into the cytochrome c oxidase (CCO) molecule, and (4) be transferred to the structures of the Golgi apparatus with antioxidant protein 1 (ATOX1), acting as a chaperone for ATP-ase 7B). ATP7B, a protein located in membranes of Golgi apparatus, transports copper to the bile ducts for excretion in feces (with participation of the MURR1 protein, reannotated to “copper metabolism MURR1 domain-containing protein 1”, COMMD1) and enables incorporation of copper into apoceruloplasmin (apoCp) to produce mature ceruloplasmin (CPN) (the main copper transport protein in the peripheral blood) and SCO 1/2 (cytochrome c oxidase assembly protein 1/2).

In the cell, copper is bound by one of the many chaperones that deliver copper to the mitochondria, to the Golgi structures, or to the cytosol, where copper can be incorporated into many cuproproteins, including clotting factors V and VIII, tyrosinase, and lysyl oxidase. Excess copper in the cell can be bound by metallothioneins. From the enterocytes, copper passes into circulation through the ATP7A protein. In a related form, this element along with blood is distributed throughout the body. Copper enters hepatocytes via the copper transporter located in the CTR1 membrane. Copper is incorporated into hepatocytes in the structures of copper-dependent proteins (cuproproteins). This process is mediated by a number of accompanying proteins. The chaperone ATOX1 transports copper ions to ATPases: ATP7A and ATP7B. Copper, which is not used in the process of enzyme synthesis, remains in the cytoplasm of hepatocytes in a form bound to metallothioneins or glutathione. In the liver, which is the main organ responsible for the metabolism of copper and which stores the most of this element, a significant part of copper is incorporated into the ceruloplasmin molecule (in an ATP7B-dependent process); in this form, it is released into the circulation.

## 5. Copper Brain Metabolism

Despite its key role, knowledge of Cu transport to the brain, its distribution, and homeostatic regulation remains limited [13]. The mechanism of copper transport to the brain and the relative role of the blood–brain barrier (BBB) and the blood–cerebrospinal fluid barrier (BCB) in regulating Cu homeostasis in the brain are not fully understood. Little is known about the transport and distribution of Cu in various regions of the brain. It was documented that Cu is transported to the brain as free copper ion, which crosses the brain’s barriers and is then distributed to the brain parenchyma and the cerebrospinal fluid. BBB appears to serve as the main input for Cu to access the parenchyma of the brain, while BCB is likely involved in the regulation of Cu homeostasis in CSF. Both the BBB and BCB express metal transporters involved in Cu transport, and the levels of their gene expression are higher in brain barrier cells than in the parenchyma of the brain. The choroid plexus (CP) showed the greatest ability to obtain Cu from the bloodstream. This structure sequesters copper and regulates the movement of copper into the cerebrospinal fluid. Observations suggest that copper ions in the brain’s capillaries can be transported more easily into the parenchyma than Cu in the CP into the cerebrospinal fluid. Several transporters can carry copper across the cell membrane; these include Ctr1, DMT1, ATP1A, and ATP7B [51,76]. The data show that all these putative copper transporters are expressed deeper in brain barrier fractions (brain capillaries and CP) than in the brain parenchyma, suggesting a possible involvement of these transporters in brain copper uptake. Since there is no barrier between the interstitial fluid and the interstitial fluid, it is proposed that the copper in the cerebrospinal fluid may be derived from copper in the mass flow of the interstitial fluid.

The copper ions enter the BBB into the space around the capillary via astroglia [45,77,78]. In addition to trading in complexes with various neurotransmitters and carrier peptides, there is also a possibility of copper uptake by autophagy as part of the protein load of extracellular vesicles [13,79].

In a normal brain, astrocytes take up copper via the Ctr1 transporter. However, the observation of the inhibitory effect of zinc on copper accumulation suggests the existence of an additional mechanism that may contribute to copper transport and its uptake into astrocytes [79]. Such a mechanism may involve the involvement of DMT1, which is able to mediate copper transport across biological membranes [80]. DMT1 is expressed in brain capillary endothelial cells, neurons, and CP epithelial cells [45]. It has been shown that both metal transporters CTR1 and DMT1 carry preferentially monovalent copper (Cu^+^) [81]. For this reason, Cu^+^ delivery to cellular uptake depends on extracellular Cu^2+^ reduction, a process that may be performed by small molecule reducing agents such as ascorbate, which has been shown to be released by astrocytes in culture [82]. It seems possible that any cytochrome b family ectocreductase or Steap protein may be involved in copper uptake, as cultured astrocytes accumulate copper rapidly upon Cu^2+^ administration to cells [83,84]. Another protein candidate that may contribute to astrocyte copper uptake appears to be prion-associated protein (PrP). PrP has been documented to be able to bind copper with low micromolar affinity, and it has been suggested to serve as a receptor for cellular copper uptake or efflux [85,86,87]. However, the role of PrP in copper homeostasis in the brain is not fully understood.

Upon import, copper Cu^+^ is chaperoned to several copper stores by specific proteins including the chaperone for copper zinc superoxide dismutase, which delivers copper to Cu,Zn-SOD [39,88], a chaperone for cytochrome c oxidase Cox17 which delivers copper to mitochondria, and antioxidant protein 1 (Atox1), which delivers copper to specific enzymes, molecules, the nucleus, and endoplasmic reticulum and to the P-type copper-transporting ATPases: ATP7A and ATP7B. ATP7 A/B, besides their significance in overall copper efflux and balance, play a critical role in copper transport between intracellular compartments [23,89,90,91,92]. Copper is released from astrocytes via ATP7A to supply neurons with copper [79].

## 6. Abnormal Copper Homeostasis—Neurodegenerative Disease

In light of its essential role for life, alterations in copper that can lead to higher or lower levels can have detrimental consequences. Research results indicate that both increased and decreased bioavailability of copper may be related to neurodegeneration [2].

### 6.1. Copper Excess—Neurodegeneration: Wilson’s Disease

The effects of copper overload can be easily analyzed in Wilson’s disease (WD), a genetically determined condition inherited in an autosomal recessive (OMIM # 277900) resulting from pathogenic mutations in the *ATP7B* gene encoding ATP-ase7B [93]. Mutations of *ATP7B* result in the formation of a protein that may have a shorter half-life, abnormal conformation, or incorrect localization in the intracellular space. Such a protein is also unable to properly interact with the ATOX1 partner protein [94,95,96]. As a result, ATP-ase7B is unable to properly perform the copper transport function [97,98,99]. Copper is not transferred to the structures of the Golgi apparatus, where the Cp molecule is synthesized under normal conditions [51], and is not excreted from hepatocytes into the bile ducts under conditions of excessive copper concentration in the cell [43,100]. As a result, this metal accumulates mainly in hepatocytes. When the content of copper in a cell reaches a critical value, exceeding its ability to bind with metallothioneins (MT)/glutathione (GSH), the cell creates conditions of strong oxidative stress and damage to the structures of proteins, lipids, and nucleic acids by free radicals [101,102]. Ultimately, cell damage and membrane breakdown occur. The excess of copper may lead the cell to direct the apoptotic pathway, influencing induction and translocation of nuclear and conformational changes in the p53 tumor suppressor protein [103,104]. Cu^2+^ ions also stimulate cell apoptosis by activating acid sphingomyelinase and ceramide release. After apoptotic/necrotic death of hepatocytes, copper is released into the bloodstream [105,106]. Circulating copper unbound to Cp (“free” copper) is highly toxic and is easily deposited in a variety of organs, including the kidneys, cornea, and brain [107].

Neurons do not store copper, so the neuronal losses in WD are believed to be secondary and nonspecific due to the ineffective function of astroglia mediating detoxification of neurons and to the toxic action of copper ions released from astrocytes damaged by edema and degeneration. Copper ions cross the BBB to the space around the capillaries, from where they are captured by the astroglia. Damage and disturbance of astroglial function presenting a picture of “primary gliopathy” is the leading morphological basis of neurological syndromes in WD (2012). In the CNS, astroglia is a multifunctional formation of active cells that provides ionic and osmotic homeostasis in the extracellular environment of neurons necessary for their metabolism. Astrocytes oversee the development and maintenance of an effective BBB, and they have the ability to capture hydrogen ions in the area of acidosis, capture and release neurotransmitters, and are the only cells capable of eliminating ammonia in the synthesis of glutamine from glutamate [108,109]. It has been documented that, in WD, copper-capturing astrocytes in the CNS, like hepatocytes in the liver, undergo edema, proliferation, hyperplasia, and degenerative changes and transform into specific forms of giant cells with characteristic morphology [110,111,112]. Among the three forms of transformed glia, type I Alzheimer’s astroglia (AIA) and Opalski cells (Opalski astroglia, OPA) are the most characteristic of WD, while type II Alzheimer’s astroglia (Alzheimer’s astroglia) are also type II (AIIA) in acquired hepatic encephalopathies of various etiologies and sometimes also in encephalopathies unrelated to liver damage [113,114,115,116]. OPA cells, often larger than neurons, are characterized by a round or oval shape, granular cytoplasm, and no protrusion in histological staining. AIA cells are large, sometimes multinucleated, with visible lobes on histological staining. They are characterized by homogeneous eosinophilic and glial fibrillary acid protein (GFAP)-positive (characteristic marker for astrocytes) cytoplasm and a large nucleus [117,118,119]. AIIA cells are distinguished by the absence of visible cytoplasm around their large, round or oval, chromatin-deficient nuclei, with lumps of chromatin beneath the nuclear membrane. The AIIA nuclei are “naked” on histological staining, as usual immunoreacting with the acid fibrin protein glia-GFAP and immunoreactivity with protein S-100—another astroglial marker—usually reveal a limb of the cytoplasm. The differentiated immunoreactivity of AIIA with the immunohistochemical markers of astroglia suggests a loss of filamentous protein and a varying degree of astrocyte failure, while the presence of intermediate forms between AIA and AIIA indicates the plasticity of these particular forms of astroglia [77,113,117,120,121].

AIA and OPA occur in WD less frequently in the regions of cavernous and cavernous necrosis and most often on the periphery. Such a topography of changes in the area of the reticular formation of the brain stem, especially in the nuclei of serotonergic sutures, can be observed in patients with central myelinolysis of the bridge. This type of symmetric demyelination of the bridge sometimes occurs in severe forms of WD and does not differ morphologically from myelinolysis of any other etiology [122,123].

Among other brain cells, oligodendrocytes appear to be particularly sensitive to copper toxicity; water swelling of the myelin sheaths and demyelination may be one of the earliest consequences of cerebral copper overload. Therefore, MRI studies provide indirect evidence of cytotoxic edema and myelin damage in WD patients [112].

There are limited reports of neurotransmitters in WD. The concentration of homovanillic acid (HVA), 5-hydroxyindole acetic acid (5HIAA), and 3-methoxy-4-hydroxyphenylglycol (a norepinephrine metabolite) (MHPG) in the cerebrospinal fluid of people with WD is low, reflecting a generalized loss of white and gray matter [124,125]. In patients with neurological disease symptoms, there is a clear loss of striated dopamine transporters. In addition, disturbances in glucose consumption have been reported in dopaminergic brain regions of WD patients [126,127].

The majority of patients with neurological symptoms show changes in brain magnetic resonance imaging (MRI); patients with liver symptoms or even those who are asymptomatic also show some abnormalities [100]. MRI usually reveals hyperintensive T2-weighted lesions, most likely associated with cellular edema, necrosis and cystic degeneration, and glial proliferation. These lesions are most often found in the putamen, caudate, thalami, midbrain, pons, white matter, and cerebellum. Copper deposits cause hypointensity in the T2-weighed images mostly found in globus pallidus, putamens, caudate nuclei, and substantia nigra [112,128]. The most common changes in MRI are features of diffuse brain atrophy [128]. There are also cerebellar or brainstem atrophy, and lateral ventricular dilatation due to basal nuclear atrophy [128]. A pathognomonic symptom for WD is the “face of the giant panda” symptom; this is a picture associated with the occurrence on T2 images of high signal intensity in the tegmentum except for the red nucleus, with preservation of the signal intensity of the lateral portion of the pars reticulata of the substantia nigra and hypointensity of the superior colliculus [129]. Some authors found a correlation between changes in the MRI image and the severity of neurological symptoms of WD [129]. There are also studies in which the authors did not state such a correlation [129,130,131]. A correlation was also investigated between the occurrence of relevant neurological disorders such as dystonia, dysarthria, Parkinson’s symptoms, etc., with lesions located in respective brain structures. It was observed that patients with dysarthria and dystonia had more often lesions in the caudate nuclei and that patients with dysarthria and dystonia at the same time had bradykinesia, changes in the putamens. Foci in the globus pallidus have also been associated with dystonia and chorea, while changes in the thalami were associated with ataxia and tremor. Hyperintense T2WI lesions may diminish after therapy, and this change may reflect edema and gliosis. In autopsy studies, higher accumulation of copper in brain was seen compared with WD than in controls [128,129,130,131,132,133,134]. 

Transcranial B-brain ultrasound of the brain shows hyperechoogenicity of the lenticular nucleus in neurological WD manifestation [135]. This correlates with local copper content based on postmortem examinations [135,136]. In asymptomatic WD patients, the brain ultrasound examination may be present even before abnormalities are seen in the MRI [136].

In a positron emission tomography (PET) study with radioactively labeled iron in humans with WD patients, increased iron uptake by the brain was diagnosed [137].

Proton magnetic resonance spectroscopy (MRS) represents an advanced method for a noninvasive assessment of brain metabolism in vivo. Cerebral glutamine compounds (Glx) mainly include glutamine and glutamate [138]. Glutamine is an astrocytic marker, while glutamate is a neurotoxin and an excitotoxic amino acid. The Glx spectrum also includes gamma-aminobutyric acid (GABA) and glucose. Other metabolites assessed in vivo by MRS are N-acetyl aspartate (NAA), which is a neuronal marker, and creatine (Cr), often used as an internal standard against which the resonance intensity of other metabolites is determined. As mentioned before, NAA in the nervous system is a neural marker; its role is to counteract the “anion deficit” in neurons or to act as a co-transport substrate for the proposed “molecular water pump” that removes metabolic water from neurons [138]; NAA is also a precursor to the biosynthesis of N-acetylaspartylglutamate, mediated by the enzyme neuronal dipeptide, and is a source of acetate for the synthesis of myelin lipids involved in energy metabolism in neuronal mitochondria, reflecting the improvement in neuronal energy. Myo-inositol (MI), also detected in MRS, is believed to be a marker of hepatic encephalopathy, and it is the major compatible organic osmolyte responsible for counteracting increased intracellular tonicity (Cho is also believed to be an osmolyte) [138].

In newly diagnosed WD patients in both globus pallidus and thalami in all investigated WD patients, significantly decreased mI/Cr and NAA/Cr ratios and an increased Lip/Cr ratio in the pallidum were observed [139]. Analysis revealed a significantly increased Glx/Cr and Lip/Cr ratio in the thalamus. In the pallidum of neurologically impaired WD patients, Cho/Cr, Glx/Cr, and Lip/Cr ratios were higher than in control subjects and the NAA/Cr was significantly lower [139]. These brain MRS findings indicate a coexistence in newly diagnosed WD patients with neurological presentation of porto-systemic shunting changes and a neurodegenerative pattern associated with Cho, Lip, and NAA/Cr depletion. The Cho peak is considered a potential biomarker for the status of membrane phospholipid metabolism [138], so that an elevated Cho signal most likely reflects an increase in membrane turnover. In pathologies characterized by membrane breakdown, such as neurodegeneration, bound Cho moieties may be liberated into the free Cho pool [138]. These changes can be caused by free copper accumulation or can be associated with gliosis because Cho is present in high concentrations in oligodendrocytes [140]. 

In MRS, patients with WD who had improved neurological status in MRS one year after treatment in 1H-MRS showed a statistically significant increase in NAA/Cr [141]. A decrease in NAA concentrations has been observed in pathological neurodegenerative processes including loss of neurons [142]. Mitochondrial proteins appear to be a target of copper toxicity, and NAA is synthesized by neuronal mitochondria [143]. In patients with neurological symptoms with WD, functional changes in neurons affecting oxidative metabolism may result in reversible changes in relative NAA levels [141].

In MRS in heterozygous WD gene carriers, statistically significant higher ratios of Glx/Cr and Lip/Cr in 1 H-MRS in Hzc in the pallidum were found. Glx is a glial marker; the increase of Glx/Cr could correlate with glial proliferation as a result of the protective function of the astrocytes against copper and iron accumulation [139]. 

As it was documented in imaging studies, the typical brain pathology observed in WD includes bilateral lesions in the putamen, globus pallidus, caudate nucleus, and other brain regions [144]. The reason for such specific localization of neuronal damage in WD is not yet understood. It is hypothesized that the vulnerability of neurons to copper overload and toxicity may be region dependent because the substantia nigra, tail of caudate nucleus, and putamen are the brain structures characterized by the highest copper concentration in humans [145]. The differences in the density of perfused capillaries and local blood flow between brain regions are known to affect brain regional differences in chemical distribution. It deserves further explanation on whether regional differences in copper uptake reflect the diversity in capillary density, blood flow, and/or the abundance of a Cu transporter [146].

The most common neurological manifestations of WD are movement disorders including tremor, ataxia, dystonia, parkinsonism, and chorea, which are associated with dysphagia, dysarthria, and drooling. Purely asymptomatic presentation is rare, and WD patients usually present different combinations of symptoms. The onset of symptoms and their severity may change rapidly even on the same day, e.g., due to fatigue, emotional stress, and increased body temperature. As the disease progresses, new neurological symptoms develop, leading to complete immobilization and cachexia. Psychiatric symptoms appear first in 15% of WD cases and with or shortly after neurological symptoms in the remaining 25%. The most common symptoms are behavior and personality disorders. Aggression, increased sex drive, irritability, phobias, poor impulse control, emotional hyperactivity, antisocial behavior, and committing crimes are common. The most common trait of personality disorders is emotional irritability. Severe depressive, manic, or schizophrenic psychoses are less common [147,148].

### 6.2. Copper Excess—Neurodegeneration: Alzheimer’s Disease

Recently, abnormal copper homeostasis has been considered an essential component of neurodegenerative diseases including Parkinson’s disease (PD), Alzheimer’s disease (AD), Huntington’s disease (HD), prion disease, systemic lupus erythematosus (SLE), and others. The role of copper in the neuropathology of AD and PD will be discussed below.

Considerable evidence has been accumulated regarding the role of copper in AD neurodegeneration [149,150]. It was found that, in AD patients, serum copper levels were approximately 54% higher than in controls and copper among the peripheral markers of oxidative stress and trace metals, which can differentiate AD patients and healthy individuals, were in a high percentage of cases (95%). Moreover, the presence of the epsilon4 allele of the apolipoprotein E (*APOE*) gene, an important genetic risk factor for AD, is associated with higher serum copper concentrations. Impairment of plasma copper levels may determine abnormal brain copper levels in AD [151,152,153].

Copper chelating agents reverse amyloid deposits in the brain of transgenic mice and prevent oxidative stress in senile plaques (SP) and neurofibrillary tangles (NFT), supporting the important role of copper in the etiopathogenesis of AD [154,155].

The metal theory of AD predicts that copper may lead to amyloid fibril accumulation [152,156]. Several in vitro studies have shown that even low copper levels can induce A*β* aggregation [157,158]. Copper can bind with a high affinity to the amino-terminal tyrosine residue in A*β* and can induce oligomerization of A*β* by oxidative modification of the amino-terminal tyrosine residue with high affinity of A*β*, which not only promotes plaque formation but also can generate neurotoxic hydrogen peroxide H2O2 through reduction of Cu^2+^ to Cu^+^ mediated by A*β* [159,160]. High concentrations of copper have been detected in A*β* plaques in the dentate gyrus subregion of the hippocampus in a mouse AD model [161,162].

It has been documented that copper can interact with amyloid precursor protein (APP) and with tau proteins [163,164]. Interactions between copper and other proteins involved in AD, including the beta-site APP-cleaving enzyme 1 (BACE1), have also been observed [164]. BACE1 is an aspartic protease that is capable of cleaving APP in the first step of A*β* production.

Thus, BACE1, APP, and A*β* are metalloproteins that are able to bind copper and have been shown to be involved in copper brain homeostasis in experimental studies [157,165]. In the brains of TgCRND8 AD model mice, upregulation of ATP7A was reported in activated microglia surrounding A*β* plaques, which may promote Cu uptake by upregulating CTR1 expression [166]. Thus, microglia sequestration of copper may provide a neuroprotective mechanism in AD, limiting free extracellular copper available for A*β* aggregation and plaque formation [17]. Conversely, transfection of fibroblast cell lines with ATP7A resulted in loss of cellular copper and decreased APP expression, suggesting that intracellular copper deficiency induced by elevated ATP7A levels may prevent A*β* by production by downregulating APP [17,167].

Recent evidence suggests that copper involvement in AD pathology may be related to neuroinflammation. The major copper transporting protein in plasma, Cp, which is elevated in the brain and serum of AD patients, can induce proinflammatory responses in cultured microglia [17,168]. These responses include increased NO release and induction of proinflammatory status mediated by IL-1*β*, TNF, NADPH oxidase, prostaglandin E2, iNOS, and COX-2 [169]. Moreover, since Cp is a copper-containing ferroxidase [170], changes in iron metabolism are associated with misplaced copper placement and Cp levels. The iron regulating peptide, hepcidin, induced by cytokines, including IL-6, inhibits the release of iron from neurons by inducing lysosomal degradation of ferroportin, an iron exporter [171]. It has been proposed that the intracellular excess iron resulting from the aging process, which further exacerbates inflammation in AD, promotes the production of APP through the iron reactive element (IRE) in the APP promoter [172,173].

Administration of copper chelator (tetrathiomolybdate) to APP/presenilin1 (PS1) transgenic animals, an animal model of AD, significantly reduced the typical features of inflammation, inducible nitric oxide synthetase, and tumor necrosis factor protein (TNF-α) levels in the brains of AD and AD mice microglia, preventing activation of signaling by nuclear factor κB (NFκB) upon stimulation with lipopolysaccharides (LPS) [174,175]. It has been shown that interferon-*γ* (IFN-*γ*), which is secreted by natural killer (NK) cells in AD patients, stimulated the expression of ATP7A in cultured microglia and altered copper homeostasis, including copper-dependent transport of ATP7A from the Golgi apparatus to the cytoplasmic vesicles [17,166].

IFN-*γ* stimulation also increased copper uptake and increased expression of the copper importer CTR1.

Another mechanism linking copper and neurodegeneration in AD is oxidative stress. The interaction of APP or A*β* with Cu^2+^ induces reduction to Cu^+^ in vitro, promoting the production of neurotoxic H2O2. Thus, “hypermetalation” of Aβ1–42 peptide determines the redox cycles of oxidative stress and H2O2 production, A*β* oligomer formation, and plaque precipitation [160,163].

Cu–Aβ interactions can also exert toxic effects by interacting with the copper binding domain of APP to produce Cu^+^ and ROS, followed by neuronal dysfunction or death [163,176]. The site-directed redox activity of copper can cause APP fragmentation and promote amyloid peptide aggregation, thereby contributing to SP formation. Copper can also directly influence the production of peroxides. Significant reductions in peroxide levels in AD patients have been noted by reducing bioavailable copper with D-penicillamine, although it is uncertain whether clinical improvement or slowing progression may occur [163,177]. The enhancement of Cu toxicity was detected in GSH-depleted neurons. In AD, changes in glutathione peroxidase (GPx) were observed in vivo and in vitro. Changes in glutathione reductase (GR) activity were also observed in the brain in Alzheimer’s disease, and GR activity was significantly increased in cultured neurons exposed to amyloidogenic AP [176,178]. These observations confirm the role of altered GSH metabolism in the etiopathology of AD with the possible contribution of the toxic effects of copper.

### 6.3. Copper Excess—Neurodegeneration: Parkinson’s Disease

The role of copper in promoting Parkinson’s disease has been confirmed in several epidemiological studies. For example, long-term exposure to copper in the workplace is associated with an increased risk of developing Parkinson’s disease [3].

Two pathological characteristics of PD are the preferential loss of dopaminergic neurons in the substantia nigra and the presence of intracellular aggregates of proteins, called Lewy bodies, which are mainly composed of alpha-synuclein fibrils [179,180,181]. There is evidence that excess copper leads to neuronal cell death and α-synuclein aggregation [3,53]. 

The molecular mechanisms by which copper dyshomeostasis may promote PD are not fully understood, and various hypotheses have been suggested. Alpha-synuclein is recognized as an important player in the pathogenesis of PD. Alpha-synuclein is a natively unfolded protein capable of interacting with membranes adopting an α-helical conformation. Under pathological conditions, the protein concentrates in oligomers and fibrils to form toxic amyloidogenic conformations, particularly rich in α-sheet structures [182,183]. The fibrillar form of this protein is the major component of Lewy bodies, a pathological hallmark of PD. In vitro studies have shown that the presence of copper in millimolar concentrations causes the formation of partially folded amyloidogenic conformations, which are more prone to aggregation [3]. The interaction of copper ions with α-synuclein has been proposed to stabilize the partially folded conformation of the protein by reducing the electrostatic repulsion between the negative charges in this protein, which are mainly present in its C-terminal region [3,183,184]. In addition to the C-terminal binding site, a different nanomolar affinity copper binding site has been described in the N-terminal region of the protein [163]. Interestingly, it has been reported that copper accelerates the formation of α-synuclein fibrils even at physiological concentrations without changing the morphology of the fibrils [3,179,185].

Another proposed mechanism for the participation of copper in the pathogenesis of PD is the ability of free copper to bind cysteine residues in proteins [3,186]. Pretreatment of rat striatum homogenates with copper, showing marked thiol reactivity, decreased specific binding sites in dopamine D2 receptors. The administration of copper caused a 40–60% reduction in the binding of dopamine D2 receptors with [3H]-spiperone, which confirms that copper-induced thiol modifications may have functional consequences [3].

As there is consensus on the role that oxidative stress and mitochondrial dysfunction play in PD progression, another widely accepted mechanism is that copper may be involved in the pathogenesis of PD by increasing oxidative stress by catalyzing harmful redox reactions that include oxygen derivatives. That depletion of the natural low molecular weight antioxidant GSH is a very early symptom in PD. There has also been a 40–90% reduction in GSH levels in the substantia nigra tissue in patients with PD during the disease stage [187,188,189]. Thus, as in AD, altered GSH metabolism may be involved in the etiopathology of PD with the possible involvement of copper toxicity.

It seems possible that the presence of free copper ions in the brain causes oxidation of dopamine, since copper exerts a direct reactivity to this neurotransmitter [190]. Dopamine oxidation can also be mediated by copper ions associated with various ligands or peptides/proteins that are involved in the process of neurodegeneration [191]. It also appears possible that α-synuclein may play a major role in stimulating copper-induced oxidation of dopamine. This possibility is supported by experimental evidence of the inhibitory effect of the presence of copper chelating agents on the production of reactive oxygen species mediated by α-synuclein oligomers [192].

It has been documented that, in contrast to neurons containing other neurotransmitters, dopamine can make dopaminergic neurons particularly susceptible to oxidative damage [193]. After its synthesis, dopamine is almost completely sequestered inside synaptic vesicles. However, the cytosolic fraction of dopamine can undergo a spontaneous autoxidation process, which leads to the formation of reactive oxygen species and dopamine quinones [3,194,195]. Copper has been demonstrated to increase the oxidation process of dopamine, leading to a variety of potentially toxic species, such as O_2_^−^, dopamine-quinones, H_2_O_2_, and hydroxyl radicals [191]. Interestingly, the locus coeruleus and substantia nigra, where a neuromelanin—a dark pigment that is able sequester both reactive dopamine-quinones and redox-active metal ions and is considered to provide a protective mechanism preventing neurotoxicity [3,191]—is mostly found, are the brain regions with the highest levels of copper [3,191,196,197].

### 6.4. Copper Toxicity—Modifying Factors: Lessons from WD

Due to the high variability of clinical symptoms of WD, which cannot be explained by genetic determinants, including various types of mutations, ATP7B studies were conducted to identify additional factors modifying copper toxicity.

Accumulation of copper in cells, caused by impaired ATPase7B function, can stimulate the production of proinflammatory cytokines, which in turn promote further copper accumulation and enhance the cytotoxic effect of copper overload [53,186]. Copper overload can lead to cell necrosis accompanied by the development of an inflammatory response. The inflammatory process can be induced and/or potentiated by ROS, which are produced by copper in the Fenton reaction [198]. ROS are mediators of signal transduction pathways that can lead to the induction of pro-inflammatory cytokine synthesis in the cell. Conversely, proinflammatory cytokines stimulate the production of ROS and thus may increase tissue damage due to copper toxicity. In addition, copper itself can stimulate the production of pro-inflammatory cytokines by activating transcription factor NFκB; conversely, an increase in the production of proinflammatory cytokines may favor the accumulation of copper in cells.

In humans, a large interindividual variability in cytokine production under normal and pathological conditions has been observed. This variability is genetically determined; it is related to the presence of functionally significant polymorphisms of genes encoding cytokines. The individual alleles of the genes coding for cytokines are associated with an increased or decreased production of cytokines under normal and/or pathological conditions. In our studies, variants of the cytokine genes interleukin-1 (*IL1B)* C-511T and interleukin-1 receptor antagonist (*IL1RN)* VNTR (variable number of tandem repeats) polymorphism were associated with WD manifestation. The carriage of the *IL1RN* VNTR * 2 allele promoted an earlier onset of clinical symptoms of WD, especially in patients with the neuropsychiatric form of the disease [199]. The carriage of the *IL1B*-511T allele was associated with a higher concentration of copper and Cp in the serum of WD patients. We explained this relationship by the activity of IL-1β as one from the inducing factors of the so-called acute phase reaction, which is related to the increased synthesis of acute phase proteins in the liver. Cp is one of these proteins. Observation of elevated copper concentration in carriers of the *IL1B*-511T allele is probably directly related to the effect of IL-1β on the increase in Cp production. There was also an association of the *IL1RN* *2 allele with elevated serum levels of Cp. The same allele was associated with an earlier manifestation of the first WD symptoms. The effect of the *IL1RN* *2 allele on the earlier clinical manifestation of WD was significant in the group of patients with a neuropsychiatric form of the disease. The presented evidence suggests that the phenotypic effect associated with genetic variation in cytokine genes is determined by the presence of specific haplotypes characterized by the coexistence of specific variants of individual cytokine genes and not by a single locus genotype [199].

Another project aimed at identifying the factors modifying the effect of copper overload in WD concerned homocysteine (Hcys). Hcys is an amino acid (aa) formed as an intermediate product during methionine metabolism. There is a lot of evidence to suggest that this aa may play an important role in modifying effects of copper toxicity. Hcys includes a thiol group in its structure showing high affinity to copper ions. Probably, therefore, an increase in Hcys concentration may contribute to an increase in copper concentration. On the other hand, it seems that the presence of copper in the cell can stimulate the production of Hcys. It turned out that the enzyme involved in synthesis of Hcys with S-adenosyl-L-homocysteine hydrolase (SAHH) is an important cytosolic copper binding protein and that the activity of this enzyme increases with increasing copper concentration. Knowledge of the physicochemical properties of Hcys and copper suggests that they may induce a synergistic cytotoxic effect. Due to the ability to function at two levels of oxidation (Cu^2+^/Cu^+^), copper ions can participate in oxidation reactions and reduction. In the presence of Cu^2+^, Hcys can be oxidized to form highly reactive free radicals and can induce oxidative stress in the cell [200,201,202,203]. 

An important role in the metabolism of HCys plays methylenetetrahydrofolate reductase (MTHFR). Two common polymorphisms in the gene encoding MTHFR (OMIM *607093) have been identified: C677T in exon 4 (NCBI SNP ID: rs 1801133) [204] and A1298C at exon 7 (NCBI SNP ID: rs 1801131). Both polymorphisms are believed to be associated with decreased MTHFR activity and hyperhomocysteinemia (HHcys). The *MTHFR* C677T polymorphism causes substitution of an amino acid from alanine to valine (A222V) in the N-terminal catalytic domain of the enzyme. MTHFR encoded by the 677T allele has a reduced enzymatic activity (about 30% in heterozygotes and 70% in homozygotes compared to control) [205]. The *MTHFR* A1298C polymorphism results in the replacement of alanine glutamate (E429A) in the C-terminal regulatory domain. This polymorphism is believed to influence regulation of MTHFR, possibly by S-adenosylmethionine, an allosteric MTHFR inhibitor that binds to the C-terminal region. We proved that, in patients with the neuropsychiatric form of WD who have the 1298C allele, clinical symptoms manifest 6 years earlier than in patients without this allele; in turn, the diplotype 677CC/1298AA is associated with a delay of 6 years onset of neuropsychiatric symptoms of WD. Hcy has been shown to cross the blood–brain barrier. Neurons are especially sensitive to the toxic effect of Hcy because they actively capture this amino acid involving a membrane transporter. HHcy was reported by other authors in patients with various neurodegenerative diseases, including AD or with schizophrenia. It is likely that patients with WD are particularly sensitive to the neurotoxic effects of Hcy, as they accumulate copper in the brain. Copper and homocysteine interactions may enhance the process of neurodegeneration [206].

Another factor that was identified to modify the phenotypic effect of copper overload due to the pathogenic mutation of the *ATP7B* gene was the commonly studied polymorphism of the apolipoprotein E (*APOE*) gene associated with the presence of 2 single nucleotide polymorphisms (SNPs) that encode 3 ApoE isoforms: ε2, ε3, and ε4. In the general population of symptomatic patients, *APOE* genotype had no impact on the clinical form or age at initial WD manifestation. However, in female patients, the genotype associated with the presence of at least one *APOE* ε4 allele was associated with earlier by four years presentation of WD symptoms compared to the *APOE* ε3/ε3 genotype. This effect was more expressed among *ATP7B* p.H1069Q homozygotes, in which the first WD symptoms manifested on about 6 years earlier than in carriers of the wild-type *APOE* ε3/ε3 genotype [207].

In another project, we showed a reduction in the efficiency of antioxidant mechanisms in the groups of newly diagnosed patients with WD and in those treated with copper overload drugs compared to the control group. The total antioxidant potential and the concentration of glutathione were lower in the group of newly diagnosed patients compared to the values observed in the group of treated patients. Lower levels of manganese superoxide dismutase (Mn-SOD) and glutathione peroxidase (GPx) have been observed in untreated patients with neuropsychiatric disease compared to untreated patients with hepatic form. Moreover, lower GPx activity was noted in patients treated with D-penicillamine compared to patients treated with zinc sulphate. Based on the obtained results, the following conclusions were drawn: (i) in WD, the effectiveness of antioxidant mechanisms is decreased; (ii) therapy to reduce copper overload improves the antioxidant status of patients but does not normalize it; (iii) the efficiency of antioxidant protection increases after the application of the therapy, probably as a result of an increase in the content of low molecular weight antioxidants; (iv) D-penicillamine may reduce GPx activity; and (v) decreasing Mn-SOD and GPx activities may be associated with neuropsychiatric presentation of WD [208].

We also analyzed the phenotypic effects of single nucleotide polymorphisms in genes encoding large-molecule antioxidants, including cytosolic enzymes—glutathione peroxidase (*GPX1* gene, rs1050450) and manganese superoxide dismutase (*SOD2* gene, rs4880)—and a peroxisomal enzyme: catalase (*CAT* gene, rs1001179). We noticed that, in the male population, the homozygosity for the *SOD2* rs4880 T allele predisposes carriers to manifest WD symptoms at an earlier age. In our study, the homozygous *CAT* rs1001179 TT genotype was associated with onset of hepatic and neuropsychiatric symptoms of WD at a later age compared with other genotypes. We concluded that the variability within the *CAT* gene may be a significant modifier of the clinical course of WD regardless of sex while the *SOD2* genotype may affect the manifestation of WD only in men. These observations indirectly confirm the important role of oxidative stress in the pathogenesis of copper-induced tissue damage resulting in clinical manifestations of WD [209].

As bidirectional relationships exist between copper and iron metabolism, we did evaluate whether changes in copper and iron metabolism are related in WD. It was previously documented that an important role in iron metabolism is played by cuproproteins with ferroxidase activity: Cp and hephaestin (Hp). Probably, copper and iron compete in cells for binding by DMT1, which transports elements to enterocytes and other cells. An important role in the regulation of the expression of genes encoding proteins involved in iron transport (*DMT1*), Cybrd1 (cytochrome b reductase 1)), is played by the so-called hypoxia induced factor (Hif-2alpha), which is stabilized by copper. Both iron and copper are transition elements that can participate in redox reactions. We have observed that (i) the values of iron metabolism parameters in WD are gender dependent; (ii) patients with neuropsychiatric form of WD are characterized by a higher serum iron concentration and show a tendency towards higher ferritin and higher total iron binding capacity (TIBC) values compared to patients with hepatic form. This observation indicates the possible participation of iron in the pathogenesis of CNS damage in WD; (iii) in patients with WD, treatment with preparations reducing the overload of the organism with copper is associated with changes in the systemic management of copper and iron; (iv) the type of treatment used affects changes in the values of iron metabolism parameters: patients treated with D-penicillamine are characterized by lower serum iron concentration and lower TIBC value than patients taking zinc preparation; (v) *HFE* genotype concerning the two most common mutations in the human homeostatic iron regulator protein (HFE) gene: *HFE* (Online Mendelian Inheritance in Men (OMIM) * 61360): C282Y (exon 4; c.845G → A; rs1800562) and p. H63D (exon 2; c.187C → G; rs1799945) is one of the factors modifying the Wilson’s disease phenotype. In previous studies, these mutations were documented to lead to disturbances in iron homeostasis in enterocytes and increased absorption of this element; and (vi) in WD, copper accumulates equally in different parts of the brain. The recorded increase in iron levels in the dentate nucleus indicates the need for further research into brain iron accumulation in WD. [210].

## 7. Copper Deficiency and Neurodegeneration

Interestingly, human disease was described, characterized with low systemic copper and neurodegeneration. It has also been established that, in some neurodegenerative diseases, copper may play a pathological role by a twofold mechanism involving the aforementioned effects of copper toxicity occurring concomitantly with reduced intracellular bioavailability [55,128,129,130]. Such double involvement of copper was described in the pathology of AD or PD as well as other diseases [211].

### 7.1. Menkes Disease 

The most common disease associated with low systemic copper content is Menkes’ disease. It is an X-linked recessive disorder caused by mutations in the ATP7A gene encoding the copper-transporting ATP7A ATPase. In Menkes’ disease, there is a deficiency of copper in the blood, kidneys, liver, and brain due to the need of ATP7A to transport copper through the basolateral membrane of intestinal epithelial cells, while copper deposits are present in intestinal enterocytes [16]. Astrocytes, in culture, express ATP7A and release copper [212,213]. This copper export and the supply of copper to other brain cells is most likely impaired in Menkes disease. The main symptoms of the disease are muscle hypotension, hypothermia, connective tissue abnormalities, skin and hair abnormalities, and progressive neurological degeneration [214].

### 7.2. Alzheimer’s Disease

Although elevated serum free copper levels might be a risk factor for AD, there are contradicting results showing that copper may also exert beneficial effects preventing neurodegeneration [165].

The beneficial role of copper in AD is indicated by the following observations: (i) depletion of intracellular copper results in a reduction of APP gene expression [215]; (ii) copper promotes the non-amyloidogenic processing of APP and lowers the A*β* production in cell culture; it has been observed that, under conditions of low intracellular copper, APP proteolysis was shifted from non-amyloidogenic to amyloidogenic processing [157]; (iii) copper increases lifetime and decreases soluble amyloid production in APP transgenic mice; and (iv) in a clinical trial with AD patients, the decline of A*β* levels in CSF, which is a diagnostic marker, is diminished.

It is hypothesized that decreased availability of copper leads to the formation of enzymatically inactive Cp. Cp plays a role as a ferroxidase and is needed for proper iron turnover. Therefore, possibly decreased copper availability may be associated with abnormalities in iron metabolism that were documented to be related to neurodegeneration. Additionally, potentially less bioavailable “free” copper may directly decrease intracellular copper levels and impair the functions of proteins using copper as a cofactor.

Interestingly, CSF copper levels were not increased in AD as shown by a meta-analysis [72]. Instead, decreased plasma levels of copper and Cp in patients with advanced AD were described, confirming previous observations that mild copper deficiency may contribute to AD progression [216]. Thus, most likely, copper plays an important role in AD pathology through a dual mechanism of copper-induced toxic A*β* deposition, concurrently with reduced intracellular copper bioavailability [17]. More research is needed to understand why there is an apparent disorder of metal homeostasis in AD and how it can be addressed therapeutically.

### 7.3. Parkinson’s Disease

While most of the studies mentioned above associate elevated copper levels with an increased risk of PD, this association is still controversial. Recent research supports the hypothesis that decreased copper levels correlate with an increased risk of developing PD. More precisely, in PD patients, the concentrations of copper and Cp in the blood and copper atoms in the Cp molecule were lower than in age-matched healthy subjects [3]. In addition, copper levels have been shown to be lower in the most affected brain areas of PD patients compared to age matched controls, with a 35–50% reduction in copper content in the locus coeruleus and substantia nigra [3].

As it was mentioned above, by binding to Cp, copper stimulates its ferroxidase activity and participates in iron homeostasis, so it may be hypothesized that some indirect toxicity mediated by altered iron concentrations may be a consequence of low copper levels. Accordingly, aceruloplasmemia, an autosomal recessive disease characterized by Cp deficiency and caused by mutations in the *CPN* gene, is associated with iron accumulation in the pancreas, liver, basal ganglia, and retina [217]. In addition, brain iron accumulation has been reported to be associated with the loss of neurons in the same regions, and the effects appear to be associated with the ability of ferrous ions to enhance oxidative stress conditions. Interestingly, some neurological symptoms of aceruloplasminemia, including the loss of motor coordination and other motor deficits, mimic clinical symptoms typical of PD [3].

### 7.4. Copper Deficiency—Neurodegeneration: SOD1

It is hypothesized that an important link between copper deficiency and neurodegeneration may be the impaired structure and/or function of superoxide dismutase 1 (SOD1).

SOD1 immunoreactivity was detected in Lewy bodies and Lewy neurites in both the substantia nigra and locus coeruleus of the PD brains, confirming previous reports of alpha-synuclein and SOD1 co-deposition and the Lewy pathology associated with PD. The formation of amorphous SOD1 aggregates was detected to be associated with the progression of PD, linking copper deficiency to disease. These SOD1-containing aggregates displaying amorphous and spherical structures were described as lacking alpha-synuclein while containing ubiquitin, suggesting an impairment of their proteasomal degradation pathway. The density of amorphous SOD1 aggregates was increased in the substantia nigra and locus coeruleus of PD brains and was significantly more elevated than in the non-degenerating regions, suggesting an association between SOD1 aggregation and neurodegeneration [3,218,219]. As the specific activity of SOD1 was attenuated in the copper-deficient substantia nigra compared to other non-degenerating regions of the brain, a model has been proposed in which copper deficiency in the locus coeruleus and substantia nigra is associated with a reduction in copper loaded SOD1, leading to accumulation of the less stable apo-protein in amorphous aggregates and loss of the ability to protect neurons from oxidative damage [3,220].

## 8. Therapeutic Strategies to Reverse Disturbances in Brain Copper Homeostasis

Copper excess plays an important role in the etiopathogenesis of the genetic syndromes such as WD and in some neurological and neurodegenerative pathologies such as PD, AD, multiple sclerosis (MS), amyotrophic lateral sclerosis (ALS), as well as idiopathic pulmonary fibrosis, in several forms of cancer and in diabetes [221,222,223,224]. It is still controversial whether the dyshomeostasis of copper and other metals such as Fe, Mn, and Zn are the primary cause or secondary consequence of PD as well as of other neurological diseases such as Alzheimer’s disease, multiple system atrophy, dementia with Lewy bodies, ALS, Huntington’s disease, frontotemporal dementia, corticobasal degeneration, and progressive supranuclear palsy [225,226,227].

The understanding that copper dysomeostasis is involved in neurodegeneration became the basis for the hypothesis that correcting disturbed copper homeostasis could be a promising strategy for treating neurodegenerative diseases and initiated research into possible therapies to correct brain copper homeostasis and to prevent/inhibit neuronal damage. Many of the approaches proposed address the potential of astrocytes to efficiently accumulate, store, and export copper and thereby to prevent dysregulation of copper homeostasis in the brain in neurodegenerative diseases. These approaches must be very different and depend on whether the toxicity of copper is due to an increased level or a deficiency of this metal ion (or both due to different mechanisms).

A useful strategy for preventing damage from copper accumulation depends on its chelation. Chelator therapy is based on their ability to selectively binding particular atoms/ions, with the formation of a stable complex ring-like structure. Metal chelators are used as nutritional supplements in radiopharmaceuticals, growth supplements in aquaculture, cosmetics, etc. Different chelating agents have been shown to decrease copper levels by different mechanisms. 

Metal-binding compounds are able to act as chelators or ionophores. Chelators, by definition, may sequestrate metal ions, excrete from receptors into the system, and can make them biounavailable (intracellular chelation), and ionophores (extracellular chelation) in contrast usually elevate the intracellular bioavailability of metal ions. Ionophores are a class of metal-binding chelators able to transfer metal ions across membranes, creating a selective channel to particular ions most commonly in the cells [228]. An example of ionophores is clioquinol (5-chloro-7-iodo-8-hydroxyquinoline, CQ), which at the beginning seemed to act only as a chelator, able to inactivate e.g., superoxide dismutase-1, but later it turned out to act as an ionophore, which caused it to raise intracellular zinc concentrations [228]. Clioquinol is a chelator of copper, iron, and zinc. Although Cu simple chelators such as d-penicillamine or trientine have been clinically used for the treatment of Cu overload, novel thiosemicarbazone and clioquinol are lately mostly investigated as potential anticancer agents [229,230]. A novel class of thiosemicarbazone compounds (di-2-pyridylketone thiosemicarbazones) binds copper and have shown great promise in terms of their anticancer activity [231]. Both CQ and disulfiram exhibit anticancer activity via Cu ionophoric activity [229].

The first chelator introduced to WD treatment in 1948 was 2,3-dimerkaptopropranol (BAL); it was noticed that, in healthy people, administration of BAL causes an increase in urinary copper excretion [232]. After several years of observation of patients treated with this specific drug, clinical improvement was found, which should be explained by the fact that it is one of the strongest copper chelating agents [134]. In 1956, Walshe introduced d-penicillamine to WD treatment [233]. The next medicine used in WD was zinc salts, followed by triethyl tetramine and tetrathiomolibdate. D-penicillamine, triethyltetramine, and BAL belong to copper chelating compounds which, by forming complexes with copper, cause its increased excretion in urine. In turn, zinc salts block the absorption of copper in the intestines, activating intestinal and hepatic metallothionein. Tetrathiomolibdate—very rarely used in the treatment of BD—works by forming complexes with copper and proteins, used in patients with neurological symptoms to prevent deterioration [234]. Served with food, it combines with copper, which prevents its absorption. Between metals, tetrathiomolibdate forms complexes with albumin and copper in the blood; these complexes enter the liver and are excreted in the bile [234]. The main goal of copper chelating therapy in WD patients is to remove copper accumulated in tissues (de-coppering phase) and to prevent re-accumulation in the maintenance phase. D-penicilamine treatment may be responsible for patients’ neurological deterioration due to an increase of free coper ions in the blood and, after, in the brain, causes increased oxidative stress and cell damage [235,236].

Tetrathiomolybdate is a better option than trientine to use in patients with neurological WD symptoms and in reduction of the number of patients with neurodegenerative disease [234]. Despite the potential efficiency and limited toxicity, the clinical use of tetrathiomolibdate is still limited by instability of the ammonium formulation or low compliance. Because of these limitations, bis-choline-tetrathiomolybdate has been recently introduced and a multicenter phase II study has been performed. The study demonstrated the efficiency of investigated drug with no drug-related neurological worsening [237]. A phase III study comparing bis-choline tetrathiomolibdate to other copper chelating compounds started in 2018 [237].

The chelating agents were also used in other neurological disorders with neurodegeneration. Their affinity towards the overload of metal ions should be as high; their metal complexes should not display any toxicity; and no negligible side effects should be seen. It has been suggested that, in AD, copper imbalance exists [238]. Cerebral amyloid in AD binds/contains increased amounts of copper and other ions such as iron and zinc compared with surrounding tissues [239]. Therefore, providing a potential focusing mechanism for metal-catalyzed pathogenetic mechanisms is thereafter an opinion that therapeutic approaches based on copper-chelating agents could be of interest in AD. D-penicillamine is a pivotal chelator used in WD treatment and administered in AD patients producing increased excretion of copper in the urine [177]. D-penicillamine also significantly decreases the content of serum peroxides [177]. The authors suggested that such a decrement may be the consequence of the prevention of copper-associated redox reactions [177]. This trial did not prove evidence of alterations in the clinical progression of AD and was terminated earlier due to adverse events.

As recently noted, intracellular copper decreases A*β* production and higher intracellular Cu levels can improve cognitive function [240], hence the great interest in new chelators-ionophores. These compounds may enhance the bioavailability of copper and zinc. Clioquinol has been used in clinical trials for the treatment of AD; this drug reduced the accumulation of beta amyloid, but in humans, it caused significant side effects [241,242,243]. Another chelator is (5,7-dichloro-2-((dimethylamino) methyl), inspired by CQ, a more effective Zn/Cu ionophore in relation to CQ, which seems to reduce insoluble A*β* levels by approximately 30% [244]. Thus, these two described compounds can cause deaccumulation of A*β* plaques loaded with Cu and Zn ions [245,246]. Unfortunately, studies in humans of cognitive improvement have been fully used in humans and have not been fully confirmed [245]. Other human clinical trials concerning 8-hydroxyquinoline concluded between 2006 and 2014 and did not show any evidence that metal-protein attenuating compounds (PBT1 or PBT2) are of benefit in AD patients [247,248,249]. Their effectiveness in improving cognitive function is still debated, and some authors believe it may be a promising therapy [246,250]. However, it should be emphasized that the abovementioned ionophores act as sophisticated compounds and that their mechanism of action may prevent the deleterious effects of breakdown in metal homeostasis and may restore metal imbalance in comparison with simple chelators [251].

Parkinson’s disease is also a neurodegenerative disorder. Alpha-synuclein is a major component of Lewy bodies and a pathogenic feature of all synucleinopathies, including PD, Lewy body dementia (DLB), and multiple system atrophy (MSA) [252]. The process of copper binding to the α-synuclein protein is an important factor in the development of PD increasing oxidative stress [253]. Recently, a study concerning copper, iron, zinc, and manganese status in PD revealed a significant decrease of copper bound to Cp with an increase of free copper that can cause oxidative stress and neurodegeneration [254]. Concentrations in the brain of postmortem PD patients compared to non-PD controls revealed diminished copper concentration in affected regions in comparison to Fe [255]. It has been therefore suggested that lowering copper contents can have some clinical value. The review recently published by Tosato et al. [227] pointed that, in PD despite copper, other metals are also deregulated in PD, including Fe, Zn, and Mn. Therefore, iron chelators, such as deferiprone used in the treatment of iron-related diseases and neurological disorders including PD, were examined [256]. The study showed that derivatives are much stronger than deferiprone in the reduction of oxidative stress and in preventing age-related insults in nerve cells [256]. The authors did show also that deferiprone and derivatives modulate several different neuroprotective signaling pathways, inhibit the activation of p38 MAP kinase and JNK kinase, and prevent the loss of PI3 kinase activity in response to toxic stress [256]. Different studies also demonstrated that deferiprone is able to chelate copper, aluminum, and zinc and to reduce their free radical formation [257]. In animal models, another metal chelator such as clioquinol played a positive effect on the suppression of PD and AD [258].

Administration of a copper chelator, cuprizone, in animal models is used as a method in modeling toxic de/remyelination of the CNS. This fact supports the idea that copper dys-homeostasis can mediate reactive oxygen species. Cuprizone can carry copper into the CNS and can cause prominent demyelination lesions through oxidative stress and oligodendrocytes toxicity [259,260]. Choi et al. found that cuprizone can also diminish activation of microglia in the spinal cord of allergic encephalopathy and can improve clinical symptoms [146].

The treatment of neurodegenerative disorders based on compounds which form complexes with metal ions such as Cu^2+^, Cu^+^, Fe^3+^, Fe^2+^, Mn^2+^, and Zn^2+^ should be further investigated. Approximately more than 250 lately tested compounds had established metal-chelating properties. In the literature, however, this type of treatment still remains under debate. 

Of the natural compounds, one of the oldest known anticancer agents with copper chelating ability is curcumin, derived from the Rhizome, Curcuma longa. The compound (found in turmeric) has well-characterized antioxidant effects in addition to a potential ability to chelate copper. The compound was shown to be protective in AD. The antioxidant cellular effects of curcumin are primarily mediated via cellular kinase signaling pathways, including JNK, Akt, and Nrf2-dependent mechanisms. Additionally, curcumin also improves numerous indicators of mitochondrial function, from caspase activation to membrane depolarization. Aside from direct ROS inactivation, as reported for peroxynitrite, curcumin may also detoxify ROS through its metal-binding and ionophore properties, which can be modulated by alteration of the aromatic ring substituents. Copper-chelated curcumins and curcuminoids have also found application for the treatment of neurodegeneration via dual-functional pro- and antioxidant effects [261,262]. Recently, various therapeutic strategies were used or considered to treat diseases that are associated with impaired copper homeostasis in the brain. The only treatment currently available for Menkes’ disease is parenteral administration of copper compounds, which may improve clinical outcomes if introduced soon after birth [92,212,214]. A better therapeutic response is obtained in newborns with Menkes’ disease, showing albeit residual ATP7A activity, who were more likely to respond to this treatment than newborns with complete loss-of-function mutations [92]. More recently, the addition of the ATP7A gene targeting the brain in a Menkes disease mouse model has shown promising results [263]. Therefore, gene therapy that restores at least a low level of functional ATP7A may become an alternative treatment option in the future [92,263].

Such a treatment can restore the insufficient ATP7A-dependent copper efflux from astrocytes as well as ATP7A-dependent copper distribution in neurons. Low levels of bioavailable copper are associated with neurodegeneration, and recent experimental evidence supporting this hypothesis suggests that the use of molecules capable of delivering copper to the brain may have a beneficial effect on disease progression. Moreover, the chemical compound containing copper diacetylbis (N (4)-methylthiosemicarbazonato) Cu^2+^ (Cu^2+^ATSM) has recently been already described as an important therapeutic strategy towards ALS. Cu^2+^-ATSM is an orally administered blood–brain barrier-permeable molecule that has previously been traditionally used in cellular imaging experiments to selectively label hypoxic tissues. It is a molecule that is additionally able to accumulate in cells with mitochondrial dysfunction, and interestingly, it accumulates in the striatum of PD patients with an accumulation level that positively correlates with the severity of the disease. It is also worth noting that, in genetic murine PD models, Cu^2+^ATSM has been shown to save dopaminergic cell loss and to improve motor dysfunction. These previously cited examples of positive results, along with the ability of Cu^2+^ATSM to accumulate in damaged tissues in PD, the abovementioned alleged role of SOD1 in the pathogenesis of PD, and the fact that Cu^2+^ATSM is able to stimulate SOD1 activity support the clinical trial I phase based on the application of Cu^2+^-ATSM, which is currently underway (NCT03204929) [3].

The protective effects of Cu^2+^-ATSM have been demonstrated in several mouse models of ALS, where the molecule has also been shown to restore the functionality of copper-deficient SOD1 [3]. It was documented that copper delivery therapy with the drug Cu^2+^-ATSM acting as an agent which releases copper into oxidative tissues could promote the survival of animal ALS models [264]. Administration of Cu^2+^-ATSM in SOD and CCS mice may reduce mortality and motor neuron deficit in symptomatic mice [265]. 

The very positive readouts led to clinical evaluation of Cu^2+^-ATSM, which is now used in one phase 2/3 clinical trial (NCT04082832).

Various copper chelators introduced in WD treatment such as tetrathiomolybdate, d-penicillamine, trientine, and lipophilic metal chelators were examined in SOD1 mice [266,267,268]. Mentioned chelators may remove copper accumulation and inhibit the peroxidase action of SOD1, which can finally postpone disease progression [266,267,268,269].

Copper histidine supplementation was not found to improve cognitive function in AD; however, it was associated with a stabilizing effect on the levels of Ab42, a biomarker of Alzheimer’s disease in cerebrospinal fluid [152,270]. A randomized, double-blind, placebo-controlled trial in AD patients using PBT2, a copper/zinc ionophore, showed the release of copper and zinc ions from senile amyloid plaques and facilitated their reuptake into cells, and it has been shown to improve cognitive function [271,272].

Therefore, it seems that the correction of disturbed copper homeostasis in the brain is a highly promising treatment strategy for neurodegenerative disorders. Due to the fact that astrocytes have the potential to efficiently accumulate, store, and export copper, their involvement in copper metabolism in the brain should be considered in all therapies aimed at preventing dysregulation of copper homeostasis in the brain in neurodegenerative disorders. Astrocytes are metabolically linked to neurons. In particular, it has been suggested that the release of lactate and GSH from astrocytes provides neurons with an important energy substrate and precursors for GSH synthesis, respectively. In copper-treated astrocytes, both lactate and GSH export are accelerated, and the excess cellular copper seems to activate mechanisms that increase the supply of lactate and GSH precursors, which may be beneficial to neighboring neurons. Increased GSH release by astrocytes has been shown to protect co-cultured motor neurons from the toxic effects of overexpression

The hSOD1 mutant linked to ALS [273]. Accelerated astrocytic release of lactate and GSH, in contrast, can also be detrimental, as evidenced by the impaired viability of neurons co-cultured with astrocytes that were stimulated with aggregated forms of amyloid-b [274]. Copper-induced alterations in the metabolic linkage between astrocytes and neurons and the ability of astrocytes regulating copper homeostasis in the brain will make astrocytes an interesting therapeutic target in diseases that are associated with disturbances in copper metabolism in the brain.

## 9. Conclusions

Copper is essential to brain cells as a cofactor and a structural component of various enzymes involved in important biochemical pathways such as the respiratory chain, antioxidative defense, and iron metabolism [275]. However, excess copper in cells is detrimental, since redox active copper can catalyze the production of hydroxyl radicals in a Fenton-like reaction, thus inducing oxidative stress and cell damage [276]; it can also affect the processes of protein aggregation or neuroinflammation. Thus, tight regulation of cellular copper metabolism is required to ensure that copper is sufficiently available for essential enzymes without toxic effects caused by its excess. This knowledge opens up an important new area for potential therapeutic interventions based on copper supplementation or removal in neurodegenerative diseases including Wilson’s disease, Menkes disease, Alzheimer’s disease, Parkinson’s disease, and others. However, much remains to be discovered, in particular, how to regulate copper homeostasis, how to prevent neurodegeneration, when to chelate copper, and when to supplement it.

## Figures and Tables

**Table 1:** Average concentration of copper in human organs (according to [13]).

Organ	Average Concentration of Copper FAAS µg/g Wet Tissue	
	Sumio et al. (1975)	Lech and Sadik (2007)
Liver	9.9	3.47
Brain	5.1	3.32
Heart	3.3	3.26
Kidney	2.6	2.15
Intestines	2.1	1.54
Lung	1.3	1.91
Spleen	1.2	1.23

FAAS, flameless atomic absorption spectrometry.

**Table 2:** Average concentration of copper in different brain areas [13].

Brain Area	Average Concentration of Copper in Descending Order FAAS (µg/g Dry Tissue)
Olfactory bulb	27.92
Caudate nucleus (tail)	23.12
Calcarine cortex	23.07
Occipital pole	21.69
Mammillary bodies	19.65
Frontal pole	18.95
Postcentral gyrus	18.83
Caudate nucleus (body)	18.46
Inferior colliculus	17.92
Optic nerve	17.79
